# What do DNA methylation studies tell us about depression? A systematic review

**DOI:** 10.1038/s41398-019-0412-y

**Published:** 2019-02-04

**Authors:** Muzi Li, Carl D’Arcy, Xintong Li, Tieyuan Zhang, Ridha Joober, Xiangfei Meng

**Affiliations:** 1 Department of Psychiatry, Faculty of Medicine, McGill University, Montreal, QC Canada; 2 School of Public Health, University of Saskatchewan, Saskatoon, SK Canada; 3 Department of Psychiatry, College of Medicine, University of Saskatchewan, Saskatoon, SK Canada; 4 Mitacs Globalink, Montreal, QC Canada; 5 Douglas Mental Health University Institute, Montreal, QC Canada

## Abstract

There has been a limited number of systematic reviews conducted to summarize the overview of the relationship between DNA methylation and depression, and to critically appraise the roles of major study characteristics in the accuracy of study findings. This systematic review aims to critically appraise the impact of study characteristics on the association between DNA methylation and depression, and summarize the overview of this association. Electronic databases and gray literatures until December 2017 were searched for English-language studies with standard diagnostic criteria of depression. A total of 67 studies were included in this review along with a summary of their study characteristics. We grouped the findings into etiological and treatment studies. Majority of these selected studies were recently published and from developed countries. Whole blood samples were the most studied common tissues. Bisulfite conversion, along with pyrosequencing, was widely used to test the DNA methylation level across all the studies. High heterogeneity existed among the studies in terms of experimental and statistical methodologies and study designs. As recommended by the Cochrane guideline, a systematic review without meta-analysis should be undertaken. This review has, in general, found that DNA methylation modifications were associated with depression. Subgroup analyses showed that most studies found *BDNF* and *SLC6A4* hypermethylations to be associated with MDD or depression in general. In contrast, studies on *NR3C1*, *OXTR*, and other genes, which were tested by only few studies, reported mixed findings. More longitudinal studies using standardized experimental and laboratory methodologies are needed in future studies to enable more systematical comparisons and quantitative synthesis.

## Introduction

A number of systematic reviews on susceptible genes and gene–environment interplay provide a comprehensive list of putative genetic and environmental risk factors for depression^1–6^. In contrast, there has been little compilation of our knowledge of DNA methylation modifications and depression.

To our knowledge, there are five reviews, including only one systematic review so far on the relationship between DNA methylation and depression^7–11^. Generally, they suggested that altered DNA methylations may be associated with the etiology of depression. Lockwood et al. in their narrative review of epigenetic findings in both animal and human studies concluded that epigenetics could play an important role in depression and suicide in humans^7^. Again, Uddin et al^8^., using a similar approach, studied the role of sex in DNA methylation and post-traumatic stress disorder and major depressive disorder (MDD), and suggested that sex differences in DNA methylation among those genes known to influence brain development may explain the sexually dimorphic risk for developing post-traumatic stress disorder and MDD. Another narrative review found the inverse association between adverse environmental factors, i.e., early-life stress, and the epigenetic modification of gene expression^9^. A review examined the association between DNA methylation of seven candidate genes and depression, and found that brain-derived neurotropic factor (*BDNF*) and nuclear receptor subfamily 3 group C member 1 (*NR3C1*) gene methylation levels may be related to depression, whereas the relationship between serotonin transporter gene (*SLC6A4*; synonyms: 5-*HTT* and *SERT*) and depression was inconsistent^11^. One recent systematic review assessed both animal and human studies and identified the correlation between burnout/depression and global and candidate-gene DNA methylation^10^. However, this review did not examine the influence of experimental and statistical methodologies and analyses on findings.

Although a few reviews are published to explore the relationships between DNA methylation modifications and depression, there has been no review critically examining experimental methodologies and verification of laboratory testing in humans. The experimental methodologies and laboratory testing are closely linked with the accuracy of results. In addition, these reviews only focused on some aspects; for example, exploring the roles of sex and stress in this relationship. In this review, we aimed to (1) systematically synthesize the major findings on DNA methylation and depression, (2) compare the similarities and differences across different studies, including experimental and laboratory factors and statistical analyses used, which might partially explain some inconsistencies in the results, and (3) discuss the challenges and opportunities for future studies.

## Materials and methods

**Fig. 1:**
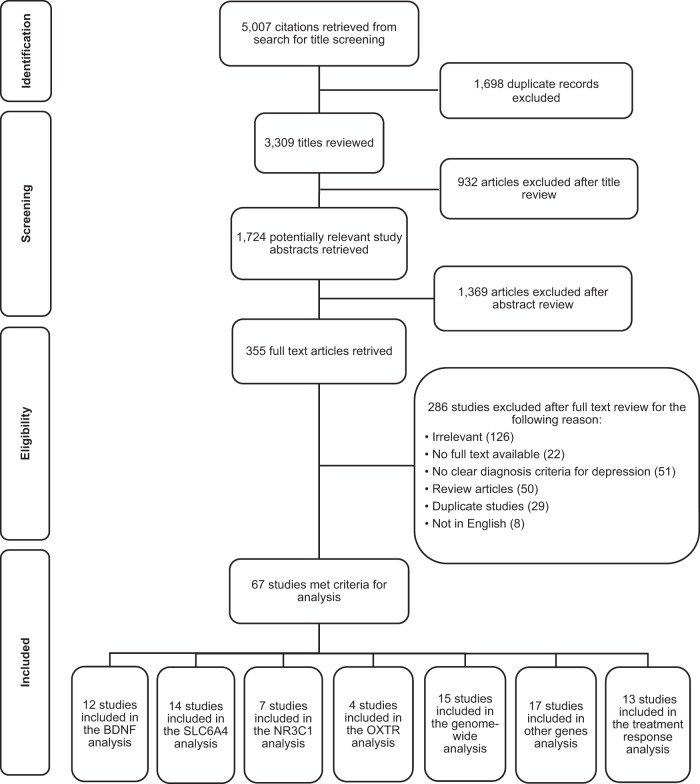
PRISMA flow diagram: DNA methylation and depression. Some selected studies had more than one study topic (i.e., *BDNF*); therefore, the total of these subgroups were bigger than the final number eligible for the review

The processing and reporting of the results of this systematic review were guided by the Preferred Reporting Items for Systematic Reviews and Meta-Analyses (PRISMA) guidelines 2009 revision ^12^. To ensure a thorough and systematic review of the literature, two methods were used to retrieve all the studies on relevant topics. We conducted a search of the computerized bibliographic databases PubMed, Web of Science, EMBASE, Medline, and Cochrane Library. The search strategy is detailed in Supplementary Appendix 1. The literature search comprised articles published until December 2017. A snowball technique was then applied to identify further studies. In addition, we manually searched other resources for other relevant studies. The reference lists of selected articles, review articles on relevant topics, and the gray literatures were screened. Figure 1 presents the process of study selection.

All suitable articles were evaluated with regard to their internal validity based on the four selection criteria as follows: (1) if they used a clear diagnosis criteria for depression (e.g., depression in general, major depressive disorder, depressive symptoms, or other types of depression), specifically the Diagnostic and Statistical Manual of Mental Disorders, 4th Edition (DSM-IV) and its updates^13^, and the International Statistical Classification of Diseases and Related Health Problems, 10th revision (ICD-10)^14^ or other generally accepted diagnostic criteria; (2) if they examined the association between DNA methylation and depression; and (3) if they provided a statistical indicator (i.e., coefficient) or original data to estimate the relationship between DNA methylation and depression. Articles were excluded if (1) they did not specify a clear diagnosis for major depression, major depressive disorder, unipolar depression, or other types of depression or (2) they were not written in English.

Two authors (M. Li and X. Li) independently screened all the retrieved articles. Inconsistencies in interpretation were resolved through group discussions (X. Li, M. Li, and X. Meng). Endnote and RefWorks were the bibliographic softwares used. Data on author(s), year of publication, sample size, study design, study cohort, experimental methods, type of tissues, candidate genes or genome, DNA purification method, DNA methylation method, DNA methylation validation, genotyping, gene expression, experimental factors, statistical methods, and major findings were extracted independently. For those studies with multiple reports, a single record denoted one study with the information extracted from multiple reports. Group discussions dealt with all the inconsistent interpretations. The reviewers endeavored to contact the original authors of the studies for any missing information in order to gather complete and consistent study information. Open-ended questions were used to prevent misleading answers.

Because of the divergence of candidate genes and genomes, for example, some studies used the candidate-gene approach and others examined the whole genome, we grouped the summarized findings according to the number of studies available, including etiological studies and treatment studies. The etiological studies were then further divided into the following subgroups, including (1) *BDNF*, (2) *SLC6A4*, (3) *NR3C1*, (4) oxytocin receptor (*OXTR)*, (5) other genes, and (6) genome-wide. Some articles were involved in multiple separate analyses as their data permitted.

## Results

A total of 67 articles met our eligibility criteria. Figure 1 shows the detailed information of the process of study selection. Table 1 presents a summary of study characteristics of these selected studies. Supplementary Appendix 2 provides a list of the references for all the selected articles corresponding to their order in Table 1. Most of the reviewed articles were published between 2014 and 2017, especially in the past 4 years. The selected studies mainly focused on adults and seniors (58/67), covering a total number of 11,935 subjects worldwide (North America: 18/67, Asia: 21/67, Europe: 24/67, and Australia: 6/67). We also evaluated study quality, including design (study design, sample size, and subject characteristics), implementation (biological sample, DNA methylation method, purification of DNA extraction, and validation of methylation), analysis (analytical method, batch effect, genotyping, and gene expression), and interpretation of results. Most studies in this review were case–control with hospital- or general population-based cohorts. There was a wide variety in terms of sample size, ranging from 11 to 1024. Whole blood was the most commonly used biological sample analyzed by generally accepted DNA methylation methods, such as bisulfite conversion with pyrosequencing. Both parametric and non-parametric statistics were used. Importantly, most of these studies did not analyze the influence of batch effect on their results (64/67), except the three studies targeted on genome-wide variations.

**Table 1:** A summary of selected articles in this systematic review

ID	First author	Publication year	Country	Sample size	Sample characteristics	Study design	Diagnoses of depression	Biological samples	DNA methylation methods/kits	Targeted genetic locations	Markers found in genome-wide studies/ CpG sites for candidate-gene studies	Major findings
**1**	Bostrom et al.	2017	Sweden	223	Population-based adolescent cohort	Case–control	Depression in general	Whole blood	Illumina 450k	Genome-wide	The promoter region of miRNA4646 and TSS of *ZSWIM5*	Two CpG sites (cg13227623 and cg04102384) predicted depression in adolescents. cg04102384 was hypomethylated
**2**	Roy et al.	2017	USA	34	Hospital-based cohort	Case–control	MDD	Peripheral blood mononuclear cells	Immunoprecipitate the 5-methyl cytosine-enriched and qPCR	*BDNF, FKBP5, CRHBP, CRHR1, NR3C1*	Promoters, CpG islands	*BDNF, FKBP5, CRHBP, CRHR1, NR3C1* gene promoters were significantly hypermethylated in MDD
**3**	Meng et al.	2017	China	162	Hospital-based cohort	Case–control	MDD	White blood cells	Bisulfite conversion, pyrosequencing	*NET* or *SLC6A2*	Promoters, other CpG sites	There were no significant differences in DNA methylation of the *NET* gene promoter between healthy controls and patients with MDD
**4**	Kaut et al.	2017	Germany	12	Senior cases and controls	Case–control	MDD	Brain tissue	Bisulfite conversion, pyrosequencing	*PSD-95* and *GLA-1*	Promoters, CpG islands	There were no significant differences in DNA methylation of *PSD-95* and *GJA-1* between controls and cases
**5**	Ryan et al.	2017	Australia	380	Late-life MDD and controls	Case–control	MDD	Buccal cells	Bisulfite conversion, pyrosequencing	*IL-6* and treatment responses	Promoters, CpG islands	Individuals with depression (current MDD or high depressive symptoms) had lower *IL6* methylation levels at one of the four sites investigated. Antidepressant use was independently associated with higher *IL-6* methylation at the same site
**6**	Shi et al.	2017	China	161	Hospital-based cohort	Case–control	MDD	Whole blood	Bisulfite conversion, pyrosequencing	*5-HTT* or *SLC6A4*	Promoters, CpG islands	Methylation (hypo- and hyper-) at positions 4 and 5 was significantly associated with MDD
**7**	Han et al.	2017	South Korea	145	Hospital-based cohort	Case–control	MDD	Whole blood	Bisulfite conversion, pyrosequencing	*TESC*	Gene body, other CpG sites	MDD had significantly higher methylation on CpG2 position of *TESC* gene-regulating genetic variant (rs7294919) than controls
**8**	Takeuchi et al.	2017	Japan	20	Cases with best and worst treatment responses	Case–control	MDD	Whole blood	Bisulfite conversion, pyrosequencing	Genome-wide	*PPF1A4, HS3ST1*	Patients’ DNA-methylation profile at specific genes such as *PPF1A4* and *HS3ST1* was associated with individual variations in therapeutic responses
**9**	Crueanu et al.	2016	Canada	32	White Caucasians, cases and controls	Case–control	MDD	PFC brain tissue	Bisulfite conversion, quantified with EpiTYPER	*SYN2*	Promoters and gene body, CpG islands	Hypomethylation of synapsins (*SYN2*) was linked to depression
**10**	Won et al.	2016	South Korea	74	Antidepressant-free cases and controls	Case–control	MDD	Whole blood	Bisulfite conversion, pyrosequencing	*SLC6A4*	Promoter region, other CpG sites	Significant inverse correlations were observed between *SLC6A4* DNA methylation and fractional anisotropy. *SLC6A4* DNA methylation was significantly higher at CpG2 in MDD
**11**	Walker et al.	2016	Scotland	29	Members of a large family multiply affected by BD and MDD	Case–control	MDD	Whole blood	Sodium bisulphite using the EZ-96 DNA Methylation Kit, bead array using the Infinium HumanMethylation450 BeadChip	Genome-wide	Three DMR regions (promoter region of *HOXA5* for hypomethylation, 5’ end of RNF39 for hypermethylation, and promoter and first exon of *AGPAT1* and *RNF5* for hypo-methylation)	Nominally significant differences in DNA methylation were observed; altered DNA methylation was a potential mechanism for mood disorders
**12**	Osborne et al.	2016	USA	291	Derived from two prospective cohorts designed to study PPD and two cohorts from which DNA was taken long after pregnancy	Case–control	PPD	Whole blood	Illumina Human Methylation 450 (HM450) bead array for 51 women with mood disorders (existing data); bisulfite conversion pyrosequencing using PyroMark MD system for the rest of the samples	Genome-wide	No site identified	Epigenetic variation at PPD biomarker loci was likely to be associated with expression
**13**	Bustamante et al.	2016	USA	147	Lifetime MDD and controls	Case–control	MDD	Whole blood	Bisulfite conversion using EpiTect Bisulfite Kit, pyrosequencing using PyroMark Q24 Assay Design Software	*NR3C1*	Promoters, CpG islands	DNA methylation was significantly lower over CpG sites 5–13 in those with vs without MDD
**14**	Na et al.	2016	South Korea	117	Recurrent MDD and controls	Case–control	MDD	Whole blood	Bisulfite conversion, pyrosequencing, using PyroMark ID system with the Pyro Gold reagents kit (Qiagen, Valencia, CA, USA)	*BDNF* and treatment response	Promoters, CpG islands	Patients with MDD had significantly higher rates of methylation at CpG2 and CpG4 than healthy controls. No difference was found in naive or on-medication patients
**15**	Kimmel et al.	2016	USA	352	Caucasian women	Cohort	PPD	Whole blood	Bisulfite conversion by EZ DNA Methylation-Gold Kit and pyrosequencing using PyroMark MD system	*OXTR*	5’-UTR, CpG islands	CpG (cg12695586) positioned in the middle of SP1 transcription factor binding site. Its methylation had a negative correlation with PPD
**16**	Kahl et al.	2016	Germany	70	Treated MDD in patients and university announcements for controls	Case–control	MDD	Whole blood	Bisulfite conversion, PCR and sequencing. Sodium-bisulfite conversion using the EpiTect® Bisulfite Kit	*GLU1, GLU4*	Promoters, CpG islands	Increased methylation of *GLUT1* in MDD
**17**	Iga et al.	2016	Japan	57	Unmediated cases and controls	Case–control	MDD	Leukocytes	Bisulfite conversion, pyrosequencing, EpiTect Plus DNA Bisulfite Kit (Qiagen)	*SLC6A4*	Promoters, CpG islands	Mean methylation level was significantly increased in patients compared with controls, *p* = 0.04. No significant difference was found in single CpG site
**18**	Oh et al.	2015	Peripheral blood samples from Australia, The Netherlands, and UK; prefrontal cortex and sperm samples from Canada	260	Cases and matched controls	Case–control	MDD	Peripheral blood, prefrontal cortex, and sperm	Bisulfite conversion, pyrosequencing using Gold Q96 reagents, and Pyromark Q24	Genome-wide	No site identified	Hypermethylated loci were found in the white blood cells of MDD twins. The brain and the sperm showed higher proportions of hypomethylated regions in MDD patients compared with the controls
**19**	Nagy et al.	2015	Canada	121	Cases with MDD and died from suicide, and controls, not died from suicide and with no MDD	Case–control	MDD	Brain tissue	Bisulfite conversion using EpiTect Bisulfite kit from Qiagen, PCR, and sequencing	Genome-wide	115 DMRs	Significant differences (decrease) in the methylation patterns specific to astrocytic dysfunction associated with depressive psychopathology
**20**	van der Knapp et al.	2015	The Netherlands	954	Adolescents cohort	Case–control	Depression in general	Whole blood	Methylation levels analyzed using EpiTYPER method; bisulfite conversion using EZ-96 DNA Methylation Kit, followed by PCR	*NR3C1* and *SLC6A4*	Promoters, CpG islands	*NR3C1* methylation levels at NR3C1_1 were positively associated with the risk of a depressive disorder and were positively associated with depressive symptom scores at follow-up, but became non-significant when accounted for depressive symptom scores at the baseline
**21**	Melas et al.	2015	Sweden	44	Female cases and controls	Case–control	Depression in general	Saliva	Bisulfite conversion using EZ-96 DNA Methylation-Gold Kit, PCR, and sequencing	*MAOA*	Gene body, other CpG sites	Subjects with a history of depression were hypomethylated, compared to controls. Female individuals were hypermethylated at the MAOA region compared to males
**22**	Hohne et al.	2015	Germany	116	Remitted MDD and healthy controls	Case–control	MDD	Peripheral blood cells	Bisulfite conversion, PCR, and sequencing using EpiTYPER assay	*FKBP5*	Gene body, other CpG sites	Subjects with TT genotype and a lifetime history of MD had a 10% higher DNA methylation rate than healthy controls with the same *FKBP5* genotype
**23**	Choi et al.	2015	South Korea	113	MDD with a mixed history of treatment	Case–control	MDD	Whole blood	Bisulfite conversion, pyrosequencing was performed on a PyroMark ID system using the Pyro Gold reagent kit (Qiagen)	*BDNF*	Promoters, other CpG sites	There were no significant differences in the *BDNF* DNA methylation status at CpG1, CpG2, CpG3, and CpG4 between patients with MDD and healthy controls
**24**	Domschke et al.	2015	Germany	94	Caucasian case cohort with antidepressants	Cohort	MDD	Whole blood	Sodium bisulfite converted using EZ-96 DNA methylation kit, PCR, and sequencing using BigDye Terminator	*MAOA* and treatment response	Promoters and gene body, not mentioned for CpG sites	The study did not find a major influence of MAOA DNA methylation on antidepressant treatment response. However, the presently observed trend towards CpG-specific *MAOA* gene hypomethylation might potentially drive impaired antidepressant treatment response in females—larger pharmacoepigenetic studies are needed
**25**	Córdova-Palomera et al.	2015	Spain	34	Caucasian MZ twins	Twin study	Depression in general	Whole blood	Bisulfite conversion, bead array using The Illumina Infinium HumanMethylation450 (450K) BeadChip	*DEPDC7*	Gene body, other CpG sites	A hypomethylation of cg09090376 in a co-twin would be associated with an increase in his/her depressive symptom score
**26**	Reiner et al.	2015	Germany	85	Female inpatients and controls	Case–control	Depression and/or dysthymia	Leukocytes	Bisulfite conversion using EpiTect Bisulfite Kit, PCR, and sequencing using BigDye Terminator v3.1 Cycle Sequencing Kit	*OXTR*	Gene body, other CpG sites	Depressed female patients had decreased *OXTR* exon 1 DNA methylation compared to non-depressed women. Exon 1 methylation appears to be associated with depressive phenotypes, whereas exon 2 methylation was influenced by genotype rs53576
**27**	Haghighi et al.	2015	USA	120	Age- and sex-matched cases and controls	Case–control	MDD	Buffy coat of blood	Bisulfite conversion by EpiTect Bisulfite Kit, pyrosequencing using PyroMark Q96 MD	*FADS1, FADS2*, and *ELOVL5*	5’-UTR, CpG islands, and shores	MDD patients had a lower methylation in *FADS2*, but higher in *ELOVL5*
**28**	Chagnon et al.	2015	Canada	43	Women aged 65 years and plus	Case–control	Depression (major and minor) and/or anxiety	Saliva	Bisulfite conversion, pyrosequencing using Pyromark 96, except for APOE analyzed on Illumina Beadchip	*BDNF, OXTR, SLC6A4*, and *APOE*	Gene body, other CpG sites	A higher BDNF and OXTR DNA methylation was observed in subjects with anxiety/depression compared to controls
**29**	Córdova-Palomera et al.	2015	Spain	34	Twin pairs with MDD and healthy controls	Case–control twin study	MDD	Whole blood	Bisulfite conversion using Illumina Infinium HumanMethylation450 Beadchip	Genome-wide	cg01122889 (*WDR26*)	Hypomethylation in *WDR26* gene was associated with a lifetime diagnosis of depression
**30**	Bell et al.	2015	USA	545	Nested case–control study in a longitudinal cohort	Nested case–control	PPD	Whole blood	Bisulfite conversion, pyrosequencing using PyroMark Gold Q24	*OXTR*	Gene body, other CpG sites	Methylation was not significantly associated with postpartum depression
**31**	Zhang et al.	2015	China	125	MDD only, with or without suicide attempts	Case–control	MDD	Whole blood	Bisulfite conversion, methylation-specific PCR	*TPH2*	Promoters, other CpG sites	The *TPH2* promoter was methylated in 36.0% (18/50) of MDD + suicide patients, as compared with that in 13.0% (10/75) of MDD patients
**32**	Nantharat et al.	2015	Thailand	62	Untreated MDD and controls	Case–control	MDD	Whole blood	Bisulfite pyrosequencing. PyroMark LINE-1 kit (Biotage-Qiagen, Uppsala, Sweden)	*NR3C1*	Promoters, CpG islands	Hypermethylation levels at CpG7 were found in MDD in females but not in males
**33**	Kleimann et al.	2015	Germany	11	Treatment-resistant cases	Perspective cohort	MDD	Whole blood	Bisulfite conversion using EpiTect Bisulfite Kit, PCR, and sequencing using BigDye Terminator Cycle Sequencing Kit	Treatment responses on *BDNF*	Promoters, CpG islands	Remitters had a significantly lower mean promoter methylation rate than non-remitters, especially exon I
**34**	Kim et al.	2015	South Korea	969	Patients with recent acute coronary syndrome	Longitudinal	Mix of major and minor depression	Leukocytes	Bisulfite conversion using EpiTech Bisulfite Kit, pyrosequencing using PSQ 96M System	*BDNF*	Promoters, CpG islands	At baseline, a higher methylation percentage in MDD compared with no depression. Higher BDNF methylation independently associated with prevalent depressive disorder at baseline and follow-up
**35**	Kaut et al.	2015	The Netherlands	12	Recurrent MDD and controls	Pilot–replication	MDD	Postmortem brain, HIP, PFC tissue	Bisulfite conversion with a ZymoResearch bisulfite kit and Ininium Human Methylation 450K bead arrays	Genome-wide, selected genes for replication	three CpG sites on *GRIN2A*	11 genes in the hypocampus and 20 genes in the prefrontal cortex revealed differential methyaltion. In replication, *GRIN2A* was found hypermethylated in both tissues and single CpG level
**36**	Kang et al.	2015	South Korea	631	Aged 65 years and plus for cases and controls	Longitudinal	Depression in general	Leukocytes	Bisulfite conversion using EpiTech Bisulfite Kit, pyrosequencing using the PSQ 96M System	*BDNF*	Promoters, CpG islands	Higher BDNF methylation was independently associated with depression and severe depressive symptoms
**37**	Kang et al.	2015	South Korea	309	Hospital-based, all women with breast cancer undergoing breast surgery	Longitudinal	Mix of major and minor depression	Leukocytes	Bisulfite conversion using EpiTech Bisulfite Kit, pyrosequencing using the PSQ 96M System	*BDNF*	Promoters, CpG islands	A higher methylation percentage at CpG9 with depression, both 1 week and 1 year after breast cancer
**38**	Januar et al.	2015	France	1024	Aged 65 years and plus for cases and controls	Case–control	MDD	Buccal cells	Bisulfite conversion, PCR, and sequencing. Sodium-bisulfite conversion using the EpiTect® Bisulfite Kit); sequencing was performed using a BigDye Terminator v3.1 Cycle Sequencing Kit	*BDNF*	Promoters, CpG islands	Depression at baseline and chronic late life was associated with higher *BDNF* methylation
**39**	Frodl et al.	2015	Ireland	60	Cases had experienced acute depressive episodes, matched on age and sex with controls	Case–control	MDD	Whole blood	Bisulfite conversion, pyrosequencing; PyroMark Q24	*SLC6A4*	Promoters, CpG islands	MDD was not significantly associated with methylation
**40**	Booij et al.	2015	Canada	69	Adults, matched on sex and gender between cases and controls, cases not taking antipsychotics or mood stabilizers	Case–control	MDD	Whole blood	Bisulfite conversion, pyrosequencing; PyroMark Q24 Software (Qiagen) for methylation percentage at each site.	*SLC6A4*, treatment response	Gene body, CpG islands	MDD diagnosis was not significantly associated with DNA methylation. Patients with SSRIs had greater methylation
**41**	Numata et al.	2015	Japan	63	Hospital-based cases and matched controls	Case–control	MDD	Whole blood	Bisulfite conversion using EZ DNA methylation Kit (ZYMO research), Infinium Human Methylation 450 Beadchips	Genome-wide	363 (313 CGIs)	363 CpG sites demonstrated lower DNA methylation in MDD patients than in controls. 18 MDD-associated DNA methylation markers to discriminate cases from controls
**42**	Haghighi et al.	2015	USA	53	MDD and suicide cases and controls	Case–control	MDD	Whole blood	Bisulfite conversion using Illumina Infinium HumanMethylation27 BeadChip	Genome-wide	Not mentioned	Increased age-related DNA methylation perturbations in the prefrontal cortex in major depression suicide compared with nonpsychiatric controls
**43**	Tadic et al.	2014	Germany	39	MDD inpatients	Cohort	MDD	Leukocytes	Bisulfite conversion, PCR, and sequencing using BigDye Terminator v3.1 Cycle Sequencing Kit	Treatment response on *BDNF*	Promoters, CpG islands	Antidepressant treatment did not significantly affect the methylation at *BDNF* promoter IV; thus, changes in the methylation status in this DNA region seem not to be involved in the response to antidepressant treatment
**44**	Khulan et al.	2014	Finland	166	Senior cases and controls	Case–control	Depressive symptoms	Whole blood	Bisulphite conversion using EZ DNA methylation kit, bead array using Illumina methylation 450k beadchip and Infinium chemistry	Genome-wide	CpG islands, shores, and TSS	Hypomethylation was associated with depressive symptoms. The results supported that DNA methylation differences may be important in the pathogenesis of psychiatric disease
**45**	Domschke et al.	2014	Germany	94	Caucasian cases with antidepressants	Cohort	MDD	Whole blood	Sodium bisulfite converted using EZ-96 DNA methylation Kit, PCR, and sequencing using BigDye Terminator	Treatment response on *5-HTT*	Gene body, CpG islands	Hypomethylation of the *5-HTT* transcriptional control region might impair antidepressant treatment response in Caucasian patients with MDD
**46**	Kaminsky et al.	2014	USA	Not mentioned	Not mentioned	Longitudinal	PPD	Whole blood	Not mentioned	*HP1BP3* and *TTC9B*	Not mentioned	*HP1BP3* and *TTC9B* (hypermethylation) predicted PPD with an area under the receiver operator characteristic curve (AUC) of 0.87
**47**	Guintivano et al.	2014	USA	93	Caucasian women	Longitudinal	PPD	Whole blood	Illumina’s Infinium Human Methylation450 Beadchip Kit	Genome-wide	Two loci within the *HP1BP3* and *TTC9B* genes	CpG methylation levels at two loci within the *HP1BP3* and *TTC9B* genes were identified as biomarkers predictive of PPD
**48**	Tseng et al.	2014	China (Taiwan)	74	MDD cases and controls	Case–control	MDD	Leukocytes	ELISA-based for global DNA methylation profiling. MethylFlash methylated DNA quantification kit (for 5-mc), MethylFlash hydroxymethylated DNA quantification kit (for 5-hmc)	Genome-wide	Gobal methylation levels, no site mentioned	Lower levels of 5-hmc and 5-mc in severe MDD than controls, no difference among severe and remitted patients
**49**	Okada et al.	2014	Japan	100	Untreated cases or cases without a history of depressive episodes	Case–control	MDD	Whole blood	Bisulfite conversion using EZ DNA methylation kits; analyzed using a MassARRAY	*SLC6A4*	Promoters, CpG islands	The pre-treatment-methylation rate(CpG3) of *SLC6A4* is associated with therapeutic responses to antidepressants in un-medicated patients with MD
**50**	Na et al.	2014	South Korea	117	Untreated cases (no history of antidepressants)	Case–control	MDD	Whole blood	Bisulfite conversion, pyrosequencing using PyroMark ID system with the Pyro Gold reagent kit (Qiagen, Valencia, CA, USA)	*NR3C1*	Promoters, CpG islands	MDD had significantly lower methylation than healthy controls at two CPG sites (CpG3, -4)
**51**	Davies et al.	2014	UK	454 (50 twins, 354 case–control)	Monozygotic twins, discordant for depression	Twin study and case–control	MDD	Whole blood	Methylated DNA immunoprecipitation combined with ultra-deep sequencing (MeDIP-seq) (enrichment for methylated regions)	Genome-wide	Coding region of *ZBTB20* gene	Both AU and UK did not identify DMR of genome-wide significance. MDD was associated with hypermethylation on the coding region of *ZBTB20*
**52**	Carlberg et al.	2014	Austria	554	Unrelated in- and outpatients of White European origin	Case-Control	MDD	Peripheral blood mononuclear cells (PBMCs)	Bisulfite conversion using EZ-96 DNA Methylation Kit. Used methylation-specific quantitative PCR following the MethyLight protocol using SYBR green	*BDNF*, treatment response	Promoters, CpG islands	*BDNF* exon I promoter was significantly increased in MDD. Current antidepressant therapy was associated with increased methylation
**53**	Dell’Osso et al.	2014	Italy	87	Stable, pharmacological treated MDD and matched controls	Case–control	MDD	Peripheral blood mononuclear cells (PBMCs)	Bisulfite conversion, PCR, and sequencing	*BDNF*, treatment response	Promoters, CpG islands	Overall lithium and valproate tend to decrease the DNA methylation level at *BDNF* gene promoter, when compared to other classes of medications. However, within each different disorder, mood stabilizers did not seem to affect DNA methylation, suggesting that such an alteration was likely not due to treatment use
**54**	Zhao et al.	2013	USA	84	MZ twins (male veterans) for lifetime and concurrent MDD	Twin study	MDD	Leukocytes	Bisulfite conversion using EZ DNA methylation kit, pyrosequencing using PSQ 96 HS System	*SLC6A4*	Promoters, CpG islands	Variation in methylation level within the promoter region of *SLC6A4* was associated with variations in depressive symptoms. A 10% increase in the difference in mean DNA methylation level was associated with a 4.4-fold increase in the difference in BDI scores. The *5-HTTLPR* genotype did not modulate this association. The use of antidepressants did not affect the relationship between *SLC6A4* methylation and depressive symptoms
**55**	Melas et al.	2013	Sweden	174	Female cases and controls	Case–control	Depression in general	Saliva	Bisulfite conversion using EZ-96 DNA Methylation-Gold Kit, PCR, and sequencing, EpiTyper software	*MAOA*	Gene body, other CpG sites	Overall *MAOA* methylation levels were decreased in depressed females compared to controls
**56**	Byrne et al.	2013	Australia	48	Queenland twin study (discordant MDD and concordant no MDD)	Twin study	MDD	White blood cells	Bisulphite conversion, Illumina Human Methylation 450 BeadChip	Genome-wide	17 sites (6 CpG islands)	The difference in mean methylation was significant in females within discordant pairs, but not in males
**57**	Kim et al.	2013	South Korea	286	Patients with a recent ischemic stroke	Longitudinal	Post-stroke depression (both major and minor)	Leukocytes	Bisulfite conversion using EpiTech Bisulfite Kit, pyrosequencing using PSQ 96M System	*SLC6A4*	Promoters, CpG islands	Higher *SLC6A4* methylation status was independently associated with a major post-stroke depression at both baseline and follow-up
**58**	Kim et al.	2013	South Korea	286	Patients with a recent ischemic stroke	Longitudinal	Post-stroke depression (both major and minor)	Leukocytes	Bisulfite conversion using EpiTech Bisulfite Kit, pyrosequencing using PSQ 96M System	*BDNF*	Promoters, CpG islands	Prevalent, persistent, and incident PSD had a higher *BDNF* methylation status. CpG site 6 was significantly associated with incident post-stroke depression
**59**	Kang et al.	2013	South Korea	108	Patients with MDD only	Longitudinal	MDD	Leukocytes	Bisulfite conversion using EpiTech Bisulfite Kit, pyrosequencing using PSQ 96M System	*SLC6A4*, treatment response	Promoters, CpG islands	*SLC6A4* methylation status as a marker for childhood adversities among MDD, but was not associated with treatment outcomes
**60**	Bayles et al.	2013	Australia	106	Newly diagnosed or currently untreated and have not been receiving antidepressants for at least 4 weeks	Case–control	MDD	Leukocytes	Bisulfite conversion, PCR, and sequencing; EpiTYPER methylation analysis	*SLC6A2* or *NET*	Promoters, CpG islands	There were no significant differences between MDD cases and controls in terms of the pattern of methylation of the SLC6A2 promoter. Antidepressant treatment did not change the result
**61**	Zill et al.	2012	Germany	162	Caucasian cases and controls	Case–control	MDD	Leukocytes	Bisulfite conversion, PCR, and sequencing, EpiTect Bisulfite Kit	*ACE*	Promoters, CpG islands	MDD patients showed a hypermethylation pattern at all the CpG sites compared to healthy controls
**62**	Sabunciyan et al.	2012	USA	154	MDD and controls	Replication	MDD	Postmortem frontal cortex, lymphoblastoid cell lines, postmortem brain	CHARM assay platform	Genome-wide	No site identified	*PRIMA1* significantly increased the methylation in MDD in pilot, but not in replication
**63**	Uddin et al.	2011	USA	100	Lifetime depression cases and non-depressed controls	Case–control	Depression in general	Whole blood	Bisulfite conversion using EZ-96 DNA Methylation Kit, bead array using HumanMethylation27 (HM 27) DNA Analysis BeadChip	Genome-wide	21 uniquely methylated and 107 uniquely unmethylated sites with depression	Uniquely unmethylated gene sets distinguished between those with versus without lifetime depression. In particular, some processes (e.g., brain development, tryptophan metabolism) showed patterns suggestive of increased methylation among individuals with depression whereas others (e.g., lipoprotein) showed patterns suggestive of decreased methylation among individuals with depression
**64**	Fuchikami et al.	2011	Japan	38	Japanese adults	Case–control	MDD	Whole blood	Bisulfite conversion using EZ DNA methylation kit	*BDNF*	Promoters, CpG islands	Significant methylation difference was found in CpGI, but not in -IV
**65**	Olsson et al.	2010	Australia	150	Australian adolescents (cases and controls)	Case–control	MDD	Buccal cells	Bisulfite conversion, Sequenom MassARRAY EpiTyping	*SLC6A4*	Promoters, CpG islands	There was no association between depressive symptoms and either buccal cell 5-HTT methylation or 5-HTTLPR. Depressive symptoms were more common among those with elevated buccal cell 5-HTT methylation who carried a 5-HTTLPR short allele
**66**	Alt et al.	2010	The Netherlands	12	Depression and control groups matched for sex, age, brain weight, and postmortem delay	Case–control	MDD	Brain tissues	Bisulphite conversion, pyrosequencing using PyroMark ID	*NR3C1*	Promoters, CpG islands	No significant difference in methylation pattern was found between case and control groups
**67**	Philibert et al.	2008	USA	192	Lifetime MDD and controls	Longitudinal	MDD	Lymphoblast cell lines	Bisulfite conversion, methylation ratios calculated by usingMassARRAY	*SLC6A4*	Promoters, CpG islands	Greater amounts of methylation in females vs males, and a trend of higher methylation was associated with greater vulnerability of lifetime MDD

MDD = major depressive disorder, PPD = postpartum depression, PFC = prefrontal cortex, BD = bipolar disorder, HIP = hippocampus, SSRI = selective serotonin reuptake inhibitors, DMR = differentially methylated regions, PSD = poststroke depression

This review was designed to apply evidence-based approaches to summarize the findings between DNA methylation and depression. High heterogeneity was identified among the studies reviewed. The Cochrane guidelines do not recommend using quantitative methods, such as meta-analysis, to synthesize the research findings. Thus, qualitative methods were then used to summarize the overview of the research findings. We present the results in two categories based on the research objectives of these selected studies, namely etiological (genome-wide and candidate-gene) and treatment studies. Supplementary Appendix 3 provides a detailed description of each subgroup and its results.

### Etiological studies: genome-wide

Although all genome-wide studies found significant methylation modifications associated with depression, both hyper*-*and hypomethylation correlations were reported. Inconsistent results were also noted. For instance, in one study, hypermethylation was previously found in a pilot study, but was not present on its replication^15^; a significant decrease in mean methylation was observed among females, but not among males^16^; lower methylation levels were found among severe MDD patients vs healthy controls, but no difference between severe vs remitted patients^17^; and one study found both hypermethylations in some processes (e.g., brain development and tryptophan metabolism), and hypomethylations in other tissues (e.g., lipoprotein)^18^. Generally, sample sizes were not associated with study designs or major findings. However, studies with large sample sizes were more likely to use DNA purification methods and examine gene expression than those with smaller samples. Results from studies with large sample sizes are considered to be more reliable.

### Etiological studies: candidate-gene

Generally, most studies found *BDNF and SLC6A4* hypermethylation to be associated with MDD or depression. Studies on *NR3C1, OXTR*, and the rest of candidate genes, which were tested by only a few studies, reported mixed findings (hyper- and hypomethylation modifications and non-significant differences). The promoter regions and CpG islands were frequently targeted in these studies. The sample size in each group varied dramatically from 12 to 1024. Some of these studies also had gene expression for significant findings. Replications of findings were better in *BDNF* and *SLC6A4* than in other studied genes. Studies with a longitudinal study design, reliable laboratory arrays, and statistical analyses were more likely to provide robust results.

### Treatment studies

Findings in this group are more inconsistent compared to those in etiological studies. Half of the studies did not identify any significant methylation sites associated with antidepressant responses, and the rest had mixed significant findings (hyper- and hypomethylations) on different candidate genes. Again, the promoter regions and CpG islands were the major targets. This group of studies had a higher level of heterogeneity compared to other subgroups, as treatment history and stages of treatments may influence methylation modifications.

## Discussion

This review firstly explored the role of DNA methylation in depression considering both the laboratory and analytic factors that could potentially confound the findings. A total of 67 articles were included in this review. The majority of the selected studies were recently published and were from developed countries. Whole blood was the most common tissue used in these analyses. Bisulfite conversion, along with pyrosequencing, was widely used to test DNA methylation level. There was a high heterogeneity among the studies in terms of the laboratory and statistical methodologies used and study designs. Large sample size and laboratory verification (DNA purification and DNA methylation validation) are the major characteristics important for accurate results.

The findings of our study are as follows. (1) For studies using candidate-gene approaches, *BDNF, NR3C1, SLC6A4, and OXTR* genes were the most frequently studied genes. Promoters and CpG islands were the common targeted regions. Overall, most of the studies found that *BDNF and SLC6A4* hypermethylations were associated with depression. Studies on *NR3C1, OXTR*, and other candidate genes reported mixed findings in terms of methylation modification and depression. Again, promoters and CpG islands still were the focus. (2) All genome-wide studies found significant methylation sites, including hyper- and hypomethylations. (3) For studies that explored antidepressant treatment responses, their results were inconsistent as they targeted on a number of different genes and different stages of treatment. (4) Large-sample size studies were more likely to use DNA purification methods, examine gene expression in their analyses, and provide more reliable results.

### Findings on etiological genome-wide studies

All genome-wide studies reported that DNA methylation was significantly associated with depression. Hypermethylations were observed in six studies on the following genes: zinc finger and BTB domain containing 20 (*ZBTB20*), heterochromatin protein 1-binding protein 3 (*HP1BP3*), tetratricopeptide repeat domain 9B (*TTC9B*), and glutamate ionotropic receptor NMDA type subunit 2A (*GRIN2A*)^19–24^.

*ZBTB20* exists in the hippocampal neurons and cerebellum granule cells^25^, and plays a role in many processes, including neurogenesis, glucose homeostasis, and postnatal growth^26^. It may also have an impact on the development and regionalization of the human hippocampus, which has been found to be related to depression^27–29^.

Both *HP1BP3* and *TTC9B* are linked to estrogen signaling. *HP1BP3* is highly expressed in the brain and is related to a number of physical and behavioral phenotypes in mice, such as dwarfism, impaired bone mass, impaired maternal behavior, and anxiety^30,31^. Lower *HP1BP3* has been found to be associated with postpartum depression and Alzheimer’s disease in humans^21,32^. *TTC9B* has been identified to be related to gonadal hormones^33^ and may be linked to hippocampal synaptic plasticity, which is critical for hippocampal long-term potentiation and depression^34^. These markers in peripheral blood may indicate estrogen-mediated epigenetic changes in the hippocampus and in turn, potentially, raise the vulnerable phenotypes based on their actions in brain^21^.

The *GRIN2A* gene provides the instructions for making a protein called glutamate receptor subunit epsilon-1 in human encoded GluN2A, which is one of the components (subunit) of a subset of *N*-methyl-D-aspartate (NMDA) receptors. They are involved in normal brain development and are responsible for changes in the brain in response to experience (synaptic plasticity), learning, and memory^26^. Methylation modifications in *GRIN2A* may play a key role in determining the function of NMDA receptors. Generally, gene promoter-region methylation could repress the gene expression, but the methylation on gene body can be positively correlated with expression activity^35^. This suggests that the hypermethylation of the *GRIN2A* gene body may result in the overexpression of NR2A and, thus, promote vulnerability for MDD via up-regulating NMDA receptor-dependent glutamatergic signaling^36^.

Hypomethylations were also observed among depression patients on the following genes: WD repeat domain 26 (*WDR26*), the promoter region of miRNA4646, 5-hydroxymethylcytosine (5-hmc), and 5-methylcytosine (5-mc)^17,23,37–41^. Consistent with our findings on WDR26, previous studies have found that the hypomethylation of *WDR26* in depressed individuals may be related to lower gene-expression levels^42^. Additionally, the decreased blood transcription levels of *WDR26* were associated with depression-related phenotypes^42–45^. 5-mc is a methylated form of the DNA base cytosine, which could be involved in the regulation of gene transcription. Its presence is important for the maintenance of the active chromatic state and for neurogenesis at non-promoter CpG islands^46^, and is associated with stable and long-term transcriptional silencing of promoters^47^. 5-mc is also found to be involved in the critical mechanism mediating genomic imprinting. This process has been identified as a key for normal development, and its abnormal imprinting can result in disorders such as Prader–Willi, Angelman, and Beckwith–Wiedemann syndrome^47^.

5-hmc is a product of conversion of 5-mc. It is related to the regulation of gene expression, prompting DNA demethylation. The three ten-eleven translocation (TET) enzymes oxidize each step in the demethylation of 5-mc. 5-mc is first converted to 5-hmc, then to 5-formylcytosine (5fC), and then to 5-carboxylcytosine (5caC), each by TET1-3^48^. Reduced levels of TET1 and, subsequently, 5hmc cause impaired self-renewal of stem cells^49^.

Notably, inconsistent results were identified within the same studies among different subgroups; for example, different sexes^16^, processes (e.g., brain development, tryptophan metabolism, and lipoprotein)^18^, tissues (white blood cells, brain, and sperm)^50^, or between pilot and replication studies^15^.

### Findings on etiological candidate-gene studies

For candidate-gene studies, the majority (11/12) of studies on *BDNF* found *BDNF* hypermethylation were associated with cases suffering from depression. Most of the studies had relatively large sample sizes and examined DNA purification. This is consistent with the recent reviews on *BDNF* and depression. Chen et al. indicated that more than half of the studies showed an increased *BDNF* methylation in depressed patients. Bakusic et al. concluded in their review that hypermethylation was consistently found in MDD subjects across the three studies selected^10^. The *BDNF* gene provides the instructions for making a protein found in the brain and spinal cord, and promotes the survival of nerve cells (neurons). It is actively involved in the growth, maturation, and maintenance of these neurons, and in the regulation of synaptic plasticity, which is important for learning and memory^26,51^. It is reported that changes in the methylation level of the *BDNF* promoter are associated with its lower expression in the prefrontal cortex^52^ and its activity in the hippocampus in animal studies^53^. A similar decrease in *BDNF* levels was also found in the serum and plasma of MDD patients; thus, it is hypothesized that MDD is related to impaired neuronal plasticity^53^.

Positive associations between *SLC6A4* methylation modifications and depression have also been identified in many studies in this review and previous reviews^10,11^. All longitudinal studies in this review and studies with more comprehensive considerations of lab and statistical work have consistently found that depression patients had *SLC6A4* hypermethylation compared to controls. *SLC6A4* gives the instructions for making a protein in the brain that is involved in the regulation of serotonergic signaling by transporting serotonin or 5-hydroxytryptamine (5-HT) from synaptic spaces into presynaptic neurons^54^ and in the regulation of emotional behaviors^55^. The alterations of *SLC6A4* play an important role in brain development and function in humans^56^. It has been hypothesized that DNA hypermethylation may result in the reduction of *SLC6A4* expression and *5-HT* reuptake, which in turn may increase the vulnerability to affective disorders at critical stages of development^57,58^.

Findings on *NR3C1, OXTR*, and other genes were less coherent. Both hypo- and hypermethylation levels were noted in depressive patients compared to controls. No significant associations between DNA methylation on these genes and depression were also reported by some studies. Similar findings were also found by recent reviews^10,11^. *NR3C1* is the receptor to which cortisol and glucocorticoids bind. It regulates gene transcriptions and is linked to development, metabolism, and immune response^59,60^. *OXTR* is a receptor of the hormone and neurotransmitter oxytocin^61,62^. It presents in the central nervous system and plays an important role in modulating various behaviors, such as stress and anxiety, social memory and recognition, sexual and aggressive behaviors, bonding/affiliation, and maternal behavior^63–65^. We found that some of the selected studies had certain limitations in terms of the type of study design, sample size, and range of laboratory work and statistical analyses. Due to the high heterogeneity across the selected studies, this review could not provide more conclusive results on these genes in terms of relationships between DNA methylation modifications on these genes and depression.

### Findings on treatment studies

Findings of this subgroup were less consistent than those of the other two subgroups analyzed. However, this is in line with another recent review on DNA methylation, and clinical response to antidepressants in MDD patients was inadequate to provide any consistent support for such a relationship^66^. Both the increased and decreased DNA methylation levels on *SLC6A4* and *BDNF* genes were associated with the use of antidepressant medications, whereas *MAOA* methylation modification was not linked to antidepressant response. The relationship between antidepressant treatment and DNA methylation of certain genes has been reported, i.e., *BDNF* DNA methylation modification was associated with decreased gene expression, which can lead to MDD^67^. The use of antidepressants can restore the decreased *BDNF* to the normal level and alleviate depressive symptoms^53,67^. Inconsistencies across all these findings may be explained by different ethnicities, duration of treatments, and pharmacogenetic heterogeneities^68,69^. Investigations on antidepressant response should cover all the different treatment stages, since the level of DNA methylation may be altered during the treatment^70^.

### Strengths and limitations

This review synthesizes the findings on DNA methylation associated with depression and critically appraised the major study characteristics that can significantly impact this association, including study design, study population, targeted genetic variations, methylation arrays, types of tissues, DNA purification, methylation validation, appropriate statistical methods, and the consideration of downstream analyses, e.g., genotyping and gene expression.

However, there are several limitations to be noted. First, this review was designed to provide an overview of the relationship between DNA methylation and depression. Therefore, all eligible studies with a wide range of genomic coverage, i.e., targeted genes or whole genome, and different types of study designs were included. As many study characteristics were heterogeneous, no pooled results were made to simply estimate this relationship. Second, although we used subgroup analyses to synthesize homogeneous studies, different types of tissues, study designs, phenotypes of the outcome, comparison groups, analytic methods, and sample sizes can still lead to inconsistent results. Third, most of studies were cross-sectional. DNA methylation level is dynamic and potentially reversible, and can be affected by a number of environmental factors. Findings from these cross-sectional studies may not be able to reveal the true nature of this complex relationship. Finally, only English databases were searched, which may limit the comprehensiveness of eligible studies.

Overall, we found that hyper- and hypomethylations on promoter regions and CpG islands of a number of genes were significantly associated with the disease. Most of the studies applied the widely acceptable laboratory techniques and statistical analyses, which made the pooled results more likely to reach a consistent finding. Future studies should adopt longitudinal study designs to explore the dynamic change of methylation levels. To allow for a systematic comparison of studies, there should be an agreement upon the consistent set of standards involving a minimum set for the items for the execution and reporting of methylation studies similar to what is required for the reporting of clinical trials, systematic reviews and meta-analysis^12,71^. Gene expression should also be routinely added into the research to uncover how, when, and what underlying mechanisms link these identified methylation sites to depression. This would advance the field and provide a firm base for the evidence on the relationship between DNA methylation and depression.

## Supplementary information

Appendix 1 Search strategy for this systematic review

Appendix 2 Data references for selected 67 articles in this systematic review

Appendix 3 A summary of findings on etiological - candidate genes studies
