# Supplementary material for: What do DNA methylation studies tell us about depression? A systematic review
Source: Transl Psychiatry. 2019 Feb 4;9:68. doi: 10.1038/s41398-019-0412-y (PMC6362194; doi:10.1038/s41398-019-0412-y)
Supplement: Supplementary file 1 — Appendix 1 Search strategy for this systematic review [file 41398_2019_412_MOESM1_ESM.docx]

**Appendix 1**

Search strategy for this systematic review

**PubMed**

((((((((depressive disorder[MeSH Terms]) OR major depressive disorder[Text Word]) OR major depression[Text Word]) OR unipolar depression[Text Word]) OR depression[Text Word]) OR depressed[Text Word]) OR depressive[Text Word])) AND ((((DNA methylation[MeSH Terms]) OR methylation[Text Word]) OR epigenetic*[Text Word])) Filters: Humans

**MEDLINE**

(mesh(depressive disorder) OR (major depressive disorder) OR (major depression) OR (unipolar depression) OR depression OR depressed OR depressive) AND (mesh(DNA methylation) OR methylation OR epigenetic*)

**Web of Sciences**

#1 TS="DNA methylation" OR TS=methylation OR TS=epigenetic*

Indexes=SCI-EXPANDED, SSCI, A&HCI, CPCI-S, CPCI-SSH, ESCI Timespan=All years

#2 TS="depressive disorder" OR TS="major depressive disorder" OR TS="major depression" OR TS="unipolar depression" OR TS=depression OR TS=depressed OR TS=depressive

Indexes=SCI-EXPANDED, SSCI, A&HCI, CPCI-S, CPCI-SSH, ESCI Timespan=All years

#3 #2 AND #1

**EMBASE**

1 ("DNA methylation" or "methylation" or epigenetic*).mp. [mp=title, abstract, heading word, drug trade name, original title, device manufacturer, drug manufacturer, device trade name, keyword]

2 limit 1 to human

3 1 and 2

4 ("depressive disorder" or "major depressive disorder" or "major depression" or "unipolar depression" or depression or depressed or depressive).mp. [mp=title, abstract, heading word, drug trade name, original title, device manufacturer, drug manufacturer, device trade name, keyword]

5 limit 4 to human

6 4 and 5

7 3 and 6

**Cochrane Library**

#1 MeSH descriptor: [Depressive Disorder] explode all trees

#2 "major depressive disorder" or "major depression" or "unipolar depression" or "depressed" or "depression" (Word variations have been searched)

#3 #2 or #1 or "depressive" (Word variations have been searched)

#4 MeSH descriptor: [DNA Methylation] explode all trees

#5 methylation or epigenetic* (Word variations have been searched)

#6 #4 or #5

#7 #3 and #6
