# Supplementary material for: What do DNA methylation studies tell us about depression? A systematic review
Source: Transl Psychiatry. 2019 Feb 4;9:68. doi: 10.1038/s41398-019-0412-y (PMC6362194; doi:10.1038/s41398-019-0412-y)
Supplement: Supplementary file 2 — Appendix 2 Data references for selected 67 articles in this systematic review [file 41398_2019_412_MOESM2_ESM.docx]

**Appendix 2**

Data references for selected 67 articles in this systematic review

1. Bostrom AE, Ciuculete DM, Attwood M, et al. A MIR4646 associated methylation locus is hypomethylated in adolescent depression. *J Affect Disord.* 2017;**220**:117-128.
2. Roy B, Shelton RC, Dwivedi Y. DNA methylation and expression of stress related genes in PBMC of MDD patients with and without serious suicidal ideation. *J Psychiatr Res.* 2017;**89**:115-124.
3. Meng L, Chen D, Pei F, Hui R, Zheng Y, Chen J. DNA methylation in the norepinephrine transporter gene promoter region is not associated with depression and hypertension. *Clin Exp Hypertens*. 2017;**39**:539-545.
4. Kaut O, Sharma A, Schmitt I, Hurlemann R, Wullner U. DNA methylation of DLG4 and GJA-1 of human hippocampus and prefrontal cortex in major depression is unchanged in comparison to healthy individuals. *J Clin Neurosci.* 2017;**43**:261-263.
5. Ryan J, Pilkington L, Neuhaus K, Ritchie K, Ancelin ML, Saffery R. Investigating the epigenetic profile of the inflammatory gene IL-6 in late-life depression. *BMC Psychiatry.* 2017;**17**:354.
6. Shi M, Sun H, Xu Y, et al. Methylation Status of the Serotonin Transporter Promoter CpG Island Is Associated With Major Depressive Disorder in Chinese Han Population: A Case-Control Study. *J Nerv Ment Dis.* 2017;**205**:641-646.
7. Han KM, Won E, Kang J, et al. TESC gene-regulating genetic variant (rs7294919) affects hippocampal subfield volumes and parahippocampal cingulum white matter integrity in major depressive disorder. *J Psychiatr Res.* 2017;**93**:20-29.
8. Takeuchi N, Nonen S, Kato M, et al. Therapeutic Response to Paroxetine in Major Depressive Disorder Predicted by DNA Methylation. *Neuropsychobiology.* 2017;**75**:81-88.
9. Cruceanu C, Kutsarova E, Chen ES, et al. DNA hypomethylation of Synapsin II CpG islands associates with increased gene expression in bipolar disorder and major depression. *BMC Psychiatry.* 2016;**16**:286.
10. Won E, Choi S, Kang J, et al. Association between reduced white matter integrity in the corpus callosum and serotonin transporter gene DNA methylation in medication-naive patients with major depressive disorder. *Transl Psychiatry.* 2016;**6**:e866.
11. Walker RM, Christoforou AN, McCartney DL, et al. DNA methylation in a Scottish family multiply affected by bipolar disorder and major depressive disorder. *Clin Epigenetics.* 2016;**8**:5.
12. Osborne L, Clive M, Kimmel M, et al. Replication of Epigenetic Postpartum Depression Biomarkers and Variation with Hormone Levels. *Neuropsychopharmacology.* 2016;**41**:1648-1658.
13. Bustamante AC, Aiello AE, Galea S, et al. Glucocorticoid receptor DNA methylation, childhood maltreatment and major depression. *J Affect Disord.* 2016;**206**:181-188.
14. Na KS, Won E, Kang J, et al. Brain-derived neurotrophic factor promoter methylation and cortical thickness in recurrent major depressive disorder. *Sci Rep.* 2016;**6**:21089.
15. Kimmel M, Clive M, Gispen F, et al. Oxytocin receptor DNA methylation in postpartum depression. *Psychoneuroendocrinology.* 2016;**69**:150-160.
16. Kahl KG, Georgi K, Bleich S, et al. Altered DNA methylation of glucose transporter 1 and glucose transporter 4 in patients with major depressive disorder. *J Psychiatr Res.* 2016;**76**:66-73.
17. Iga J, Watanabe SY, Numata S, et al. Association study of polymorphism in the serotonin transporter gene promoter, methylation profiles, and expression in patients with major depressive disorder. *Hum Psychopharmacol.* 2016;**31**:193-199.
18. Oh G, Wang SC, Pal M, et al. DNA modification study of major depressive disorder: beyond locus-by-locus comparisons. *Biol Psychiatry.* 2015;**77**:246-255.
19. Nagy C, Suderman M, Yang J, et al. Astrocytic abnormalities and global DNA methylation patterns in depression and suicide. *Mol Psychiatry.* 2015;**20**:320-328.
20. van der Knaap LJ, van Oort FV, Verhulst FC, Oldehinkel AJ, Riese H. Methylation of NR3C1 and SLC6A4 and internalizing problems. The TRAILS study. *J Affect Disord.* 2015;**180**:97-103.
21. Melas PA, Forsell Y. Hypomethylation of MAOA's first exon region in depression: a replication study. *Psychiatry Res.* 2015;**226**:389-391.
22. Höhne N, Poidinger M, Merz F, et al. FKBP5 genotype-dependent DNA methylation and mRNA regulation after psychosocial stress in remitted depression and healthy controls. *Int J Neuropsychopharmacol.* 2015;**18**:pii: pyu087.
23. Choi S, Han KM, Won E, Yoon BJ, Lee MS, Ham BJ. Association of brain-derived neurotrophic factor DNA methylation and reduced white matter integrity in the anterior corona radiata in major depression. *J Affect Disord.* 2015;**172**:74-80.
24. Domschke K, Tidow N, Schwarte K, et al. Pharmacoepigenetics of depression: no major influence of MAO-A DNA methylation on treatment response. *J Neural Transm (Vienna).* 2015;**122**:99-108.
25. Cordova-Palomera A, Fatjo-Vilas M, Palma-Gudiel H, Blasco-Fontecilla H, Kebir O, Fananas L. Further evidence of DEPDC7 DNA hypomethylation in depression: A study in adult twins. *Eur Psychiatry.* 2015;**30**:715-718.
26. Reiner I, Van IMH, Bakermans-Kranenburg MJ, Bleich S, Beutel M, Frieling H. Methylation of the oxytocin receptor gene in clinically depressed patients compared to controls: The role of OXTR rs53576 genotype. *J Psychiatr Res.* 2015;**65**:9-15.
27. Haghighi F, Xin Y, Chanrion B, et al. Increased DNA methylation in the suicide brain. *Dialogues Clin Neurosci.* 2014;**16**:430-438.
28. Chagnon YC, Potvin O, Hudon C, Preville M. DNA methylation and single nucleotide variants in the brain-derived neurotrophic factor (BDNF) and oxytocin receptor (OXTR) genes are associated with anxiety/depression in older women. *Front Genet.* 2015;**6**:230.
29. Cordova-Palomera A, Fatjo-Vilas M, Gasto C, Navarro V, Krebs MO, Fananas L. Genome-wide methylation study on depression: differential methylation and variable methylation in monozygotic twins. *Transl Psychiatry.* 2015;**5**:e557.
30. Bell AF, Carter CS, Steer CD, et al. Interaction between oxytocin receptor DNA methylation and genotype is associated with risk of postpartum depression in women without depression in pregnancy. *Front Genet.* 2015;**6**:243.
31. Zhang Y, Chang Z, Chen J, et al. Methylation of the tryptophan hydroxylase2 gene is associated with mRNA expression in patients with major depression with suicide attempts. *Mol Med Rep.* 2015;**12**:3184-3190.
32. Nantharat M, Wanitchanon T, Amesbutr M, Tammachote R, Praphanphoj V. Glucocorticoid receptor gene (NR3C1) promoter is hypermethylated in Thai females with major depressive disorder. *Genet Mol Res.* 2015;**14**:19071-19079.
33. Kleimann A, Kotsiari A, Sperling W, et al. BDNF serum levels and promoter methylation of BDNF exon I, IV and VI in depressed patients receiving electroconvulsive therapy. *J Neural Transm (Vienna).* 2015;**122**:925-928.
34. Kim JM, Stewart R, Kang HJ, et al. BDNF methylation and depressive disorder in acute coronary syndrome: The K-DEPACS and EsDEPACS studies. *Psychoneuroendocrinology.* 2015;**62**:159-165.
35. Kaut O, Schmitt I, Hofmann A, et al. Aberrant NMDA receptor DNA methylation detected by epigenome-wide analysis of hippocampus and prefrontal cortex in major depression. *Eur Arch Psychiatry Clin Neurosci.* 2015;**265**:331-341.
36. Kang HJ, Kim JM, Bae KY, et al. Longitudinal associations between BDNF promoter methylation and late-life depression. *Neurobiol Aging.* 2015;**36**:1764 e1761-1764, e1767.
37. Kang HJ, Kim JM, Kim SY, et al. A Longitudinal Study of BDNF Promoter Methylation and Depression in Breast Cancer. *Psychiatry Investig.* 2015;**12**:523-531.
38. Januar V, Ancelin ML, Ritchie K, Saffery R, Ryan J. BDNF promoter methylation and genetic variation in late-life depression. *Transl Psychiatry.* 2015;**5**:e619.
39. Frodl T, Szyf M, Carballedo A, et al. DNA methylation of the serotonin transporter gene (SLC6A4) is associated with brain function involved in processing emotional stimuli. *J Psychiatry Neurosci.* 2015;**40**:296-305.
40. Booij L, Szyf M, Carballedo A, et al. DNA methylation of the serotonin transporter gene in peripheral cells and stress-related changes in hippocampal volume: a study in depressed patients and healthy controls. *PLoS One.* 2015;**10**:e0119061.
41. Numata S, Ishii K, Tajima A, et al. Blood diagnostic biomarkers for major depressive disorder using multiplex DNA methylation profiles: discovery and validation. *Epigenetics.* 2015;**10**:135-141.
42. Haghighi F, Galfalvy H, Chen S, et al. DNA methylation perturbations in genes involved in polyunsaturated Fatty Acid biosynthesis associated with depression and suicide risk. *Front Neurol.* 2015;**6**:92.
43. Tadic A, Muller-Engling L, Schlicht KF, et al. Methylation of the promoter of brain-derived neurotrophic factor exon IV and antidepressant response in major depression. *Mol Psychiatry.* 2014;**19**:281-283.
44. Khulan B, Manning JR, Dunbar DR, et al. Epigenomic profiling of men exposed to early-life stress reveals DNA methylation differences in association with current mental state. *Transl Psychiatry.* 2014;**4**:e448.
45. Domschke K, Tidow N, Schwarte K, et al. Serotonin transporter gene hypomethylation predicts impaired antidepressant treatment response. *Int J Neuropsychopharmacol.* 2014;**17**:1167-1176.
46. Kaminsky Z, Payne J. Seeing the future: epigenetic biomarkers of postpartum depression. *Neuropsychopharmacology.* 2014;**39**:233-234.
47. Guintivano J, Arad M, Gould TD, Payne JL, Kaminsky ZA. Antenatal prediction of postpartum depression with blood DNA methylation biomarkers. *Mol Psychiatry.* 2014;**19**:560-567.
48. Tseng PT, Lin PY, Lee Y, et al. Age-associated decrease in global DNA methylation in patients with major depression. *Neuropsychiatr Dis Treat.* 2014;**10**:2105-2114.
49. Okada S, Morinobu S, Fuchikami M, et al. The potential of SLC6A4 gene methylation analysis for the diagnosis and treatment of major depression. *J Psychiatr Res.* 2014;**53**:47-53.
50. Na KS, Chang HS, Won E, et al. Association between glucocorticoid receptor methylation and hippocampal subfields in major depressive disorder. *PLoS One.* 2014;**9**:e85425.
51. Davies MN, Krause L, Bell JT, et al. Hypermethylation in the ZBTB20 gene is associated with major depressive disorder. *Genome Biol.* 2014;**15**:R56.
52. Carlberg L, Scheibelreiter J, Hassler MR, et al. Brain-derived neurotrophic factor (BDNF)-epigenetic regulation in unipolar and bipolar affective disorder. *J Affect Disord.* 2014;**168**:399-406.
53. Dell'Osso B, D'Addario C, Carlotta Palazzo M, et al. Epigenetic modulation of BDNF gene: differences in DNA methylation between unipolar and bipolar patients. *J Affect Disord.* 2014;**166**:330-333.
54. Zhao J, Goldberg J, Bremner JD, Vaccarino V. Association between promoter methylation of serotonin transporter gene and depressive symptoms: a monozygotic twin study. *Psychosom Med.* 2013;**75**:523-529.
55. Melas PA, Wei Y, Wong CC, et al. Genetic and epigenetic associations of MAOA and NR3C1 with depression and childhood adversities. *Int J Neuropsychopharmacol.* 2013;**16**:1513-1528.
56. Byrne EM, Carrillo-Roa T, Henders AK, et al. Monozygotic twins affected with major depressive disorder have greater variance in methylation than their unaffected co-twin. *Transl Psychiatry.* 2013;**3**:e269.
57. Kim JM, Stewart R, Kang HJ, et al. A longitudinal study of SLC6A4 DNA promoter methylation and poststroke depression. *J Psychiatr Res.* 2013;**47**:1222-1227.
58. Kim JM, Stewart R, Kang HJ, et al. A longitudinal study of BDNF promoter methylation and genotype with poststroke depression. *J Affect Disord.* 2013;**149**:93-99.
59. Kang HJ, Kim JM, Stewart R, et al. Association of SLC6A4 methylation with early adversity, characteristics and outcomes in depression. *Prog Neuropsychopharmacol Biol Psychiatry.* 2013;**44**:23-28.
60. Bayles R, Baker EK, Jowett JB, et al. Methylation of the SLC6a2 gene promoter in major depression and panic disorder. *PLoS One.* 2013;**8**:e83223.
61. Zill P, Baghai TC, Schule C, et al. DNA methylation analysis of the angiotensin converting enzyme (ACE) gene in major depression. *PLoS One.* 2012;**7**:e40479.
62. Sabunciyan S, Aryee MJ, Irizarry RA, et al. Genome-wide DNA methylation scan in major depressive disorder. *PLoS One.* 2012;**7**:e34451.
63. Uddin M, Koenen KC, Aiello AE, Wildman DE, de los Santos R, Galea S. Epigenetic and inflammatory marker profiles associated with depression in a community-based epidemiologic sample. *Psychol Med.* 2011;**41**:997-1007.
64. Fuchikami M, Morinobu S, Segawa M, et al. DNA methylation profiles of the brain-derived neurotrophic factor (BDNF) gene as a potent diagnostic biomarker in major depression. *PLoS One.* 2011;**6**:e23881.
65. Olsson CA, Foley DL, Parkinson-Bates M, et al. Prospects for epigenetic research within cohort studies of psychological disorder: a pilot investigation of a peripheral cell marker of epigenetic risk for depression. *Biol Psychol.* 2010;**83**:159-165.
66. Alt SR, Turner JD, Klok MD, et al. Differential expression of glucocorticoid receptor transcripts in major depressive disorder is not epigenetically programmed. *Psychoneuroendocrinology.* 2010;**35**:544-556.
67. Philibert RA, Sandhu H, Hollenbeck N, Gunter T, Adams W, Madan A. The relationship of 5HTT (SLC6A4) methylation and genotype on mRNA expression and liability to major depression and alcohol dependence in subjects from the Iowa Adoption Studies. *Am J Med Genet B Neuropsychiatr Genet.* 2008;**147B**:543-549.
