# Supplementary material for: What do DNA methylation studies tell us about depression? A systematic review
Source: Transl Psychiatry. 2019 Feb 4;9:68. doi: 10.1038/s41398-019-0412-y (PMC6362194; doi:10.1038/s41398-019-0412-y)
Supplement: Supplementary file 3 — Appendix 3 A summary of findings on etiological - candidate genes studies [file 41398_2019_412_MOESM3_ESM.docx]

**Appendix 3**

A summary of findings on etiological - candidate genes studies

Etiological - genome-wide studies

There were 17 studies, published from 2013 to 2017, using whole-genome wide approaches to examine the relationship between DNA methylation and depression. Study sample sizes ranged from 12 to 454 subjects, and over half (52.9%, 9/17) of the studies contained relatively small numbers of sample (<100). Subjects were recruited from existing hospitals, or population-based studies or databases. The most frequently used study design was case-control (76.5, 13/17). The whole-blood sample was the common specimen for the analysis. Most studies included methylation validation procedure (64.7%, 11/17), but no DNA purification in their arrays (64.7%, 11/17). The majority (76.5%, 13/17) of these studies used bisulfite conversion for DNA methylation. Bead arrays were widely applied using Infinium Human Methylation Beadchips (64.7%, 11/17). Almost half (47.0%, 8/17) of these studies also did gene expression afterwards.

Although all these studies found significant modifications in the level of DNA methylation among depression cases, both positive (*hypermethylation*) and negative (*hypomethylation*) correlations were noted. Inconsistent results were also identified. For instance, increased methylation was previously shown in pilot study, but not present in its replication;^1^ a significant decrease in mean methylation was observed among females, but not for males;^2^ lower methylations levels were found among severe MDD patients *vs.* healthy controls, but no difference between severe vs. remitted patients;^3^ and one study found both *hyper*methylations in some processes (e.g. brain development and tryptophan metabolism), and *hypo*methylations in other tissues (e.g. lipoprotein).^4^

Generally, sample sizes were not associated with study designs and major findings. However, studies with large sample sizes were more likely to use DNA purification methods and examine gene expression than those with smaller samples. Results from studies with large sample sizes are more reliable.

Etiological studies- Candidate *gene studies- BDNF*

There were 12 articles published from 2011 to 2017, studying the relationship between DNA methylation on *BDNF* gene and depression. Their sample size ranged from 34 to 1,024 subjects. Studies used case-control (66.7%, 8/12) and short-term longitudinal cohort designs (33.3%, 4/12) were used. Subjects were from hospitals and general population. The whole blood samples were the primary choice (75%, 9/12), followed by buccal cells and saliva samples. DNA methylation was mostly tested by bisulfite pyrosequencing (66.7%, 8/12), followed by methylation-specific quantitative PCR. Most of the studies checked DNA purification (66.7%, 8/12), but did not include methylation validation (83.3%, 10/12). Seven studies also did genotyping (66.7%, 7/12), but only one recent study conducted gene expression. A half of these studies (50%, 6/12) had the relatively large sample sizes (>200), and majority of the studies with large sample size performed DNA purification (5/6).

Consistently, most studies (83.3%, 11/12) found that people with depression or post-stroke depression were more likely to have hypermethylation on multiple *BDNF* CpG sites. Only one study had a small sample size and no report on laboratory factors in their analyses, and did not replicate the above finding.^5^

Etiological studies- Candidate *gene studies- SLC6A4*

There were 13 studies examined the relationship between DNA methylation on *SLC6A4* and depression. Findings of this group are mixed, including seven studies found that *SLC6A4* hypermethylation were linked to the risk of depression, depressive symptoms, or post-stroke depression, and the other six studies did not find any association between methylation and depression. The sample sizes in this group ranged from 43 to 286 subjects, except one study had 954 subjects. Eight studies were case-control. There were three longitudinal studies with less one-year follow-ups, and one twins’ study. Subjects were from hospitals and population databases or from general population. Whole-blood sample was the primary biological sample (76.9%, 10/13), followed by buccal cells and saliva. Less than half studies tested for both DNA purification and methylation validation. More than half of studies (61.5%, 8/13) applied pyrosequencing followed by EpiTYPER or MassARRAY analysis, for DNA methylation. Most of the studies (9/13) also tested genotyping, but only four (4/13) examined gene expression.

Three longitudinal studies, which represented 63.2% of the group subjects, consistently found that *SLC6A4* hypermethylation was significantly associated with depression and depressive symptoms.^6-8^ This finding was supported by the twin’s study^9^ and a case-control study that had a most comprehensive consideration of laboratory factors and statistical analysis.^10^

Etiological studies- Candidate *gene studies- NR3C1*

Six articles studied the role of DNA methylation on *NR3C1* gene in depression. Their major findings were controversial, including both significant and non-significant relationships. Both *hypo*- and *hyper*-methylated CpG sites on *NR3C1* gene were noted. In this group of studies there was a great variation in terms of sample size, which ranged from 12 to 954 subjects. Most of studies were case-control, except for one longitudinal cohort study. Subjects were from hospital- or the general population. Whole-blood sample was the primary choice, except one study used post-mortem brain tissues. Most of studies did test DNA purification and methylation validation. Pyrosequencing, followed by EpiTYPER analysis, were used to test the level of DNA methylation. Most studies did also applied gene expression, and one even did genotyping.

Studies with longitudinal study designs, more reliable laboratory arrays and statistical analyses consistently showed that people with *NR3C1* hypermethylation were more likely to report depression and/or depressive symptoms.^8, 11^

Etiological studies- Candidate *gene studies- OXTR*

Four articles met the criteria to explore the relationship between DNA methylation on *OXTR* gene and depression. Sample sizes in this group of studies ranged from 43 to 545 subjects. Most (3/4) of them were case-control studies, and one was a longitudinal cohort study. Subjects were mainly recruited from the general population (91.7%), and the rest (8.3%) of the studies used inpatients and controls recruited from advertisements. Again, the whole blood was the primary tissue choice (75%), followed by saliva. DNA purification was tested by half of these studies. None did the methylation validation test. Bisulfite treatment and pyrosequencing were used for methylation arrays. All the studies did also test genotyping, while only one study examined gene expression.

Due to the defects in study design (i.e. small sample sizes), and lab factors (lack of DNA purification, or methylation validation), findings from these four studies are difficult to interpret. Two case-control studies had small sample sizes (N<100) found inconsistent results. The third case-control failed to apply valid lab arrays and found a non-significant association. The longitudinal cohort study with 353 subjects did not perform DNA purification or methylation validation.

Etiological studies- *Other candidate genes*

A total of 18 studies met the eligibility criteria for the relationship between DNA methylation on other candidate genes and depression. They included: *Glucose transporter 1 (GLUT1), Glucose transporter type 4 (GLUT4), Tryptophan hydroxylase 2 (TPH2), Angiotensin converting enzyme (ACE), Apolipoprotein E (APOE), Fatty acid desaturase 1 (FADS1), Fatty acid desaturase 2 (FADS2), Elongation of very long chain fatty acid elongase 5 (ELOVL5), Heterochromatin protein 1, binding protein 3 (HP1BP3), tetratricopeptide repeat domain 9B (TTC9B), FK506 binding protein 5 (FKBP5), monoamine oxidase A (MAOA), DEP domain containing 7 (DEPDC7), neurotransmitter transporter (NET or SLC6A2), Corticotropin releasing hormone receptor 1 (CRHR1), Corticotropin releasing hormone binding protein (CRHBP), postsynaptic density protein 95 (PSD-95), Glucoamylase 1 (GLA-1), Synapsin II (SYN2), and Interleukin 6 (IL-6), Tescaclin (TESC)* . *Hypermethylation* was found in depressed cases in seven studies (GLUT1, ACE, ELOVL5, FKBP5, CRHBP, CRHR1, TESC);^12-18^ in contrast, *hypomethylation* was found in depression in seven studies (FADS2, MAOA, DEPDC7, IL-6, SYN2);^14, 17, 19-23^ and no significant difference in methylation levels was found between depressed patients and their healthy controls in five studies (*GLUT4, APOE, NET, PSD-95, GLA-1*).^12, 24-26^ In addition, one study found that patients who were both depressive and suicidal had hypermethylation in the *TPH2* promoter region, as compared with in depression-only patients.^27^ Finally, one study found that methylated *HP1BP3* and *TTC9B* predicted postpartum depression. In their replication analysis, these biomarkers were able to accurately segregate postpartum depression status in women, but the prediction was in the opposite direction to the pilot analysis.^28^

This group of studies had a wide variation of sample sizes (34 to 380 subjects), and most of them had relatively small sample sizes (<100). Again, case-control was the most used study design, followed by longitudinal cohort and twins’ study. Subjects were hospital-based and general population. Two studies in this group did not mention the source of their samples. Most studies (10/15) used whole-blood for arrays, and the rest used saliva, brain tissue, buccal cells, and white cells. Most of studies tested DNA purification, but only two examined methylation validation. Consistently, DNA methylation was measured via bisulfite conversion and pyrosequencing and then analyzed by EpiTYPER. A few studies did genotyping and/or gene expression. Notably, in one study the same outcomes were obtained from the initial study on *MAOA* and its replication study using an independent sample.^19, 20^ Due to the fact that many genes were studied in this group and most studies failed to apply strong study designs, or better laboratory and analytic factors in their execution, it is hard to weigh the value of their findings.

Treatment studies

There were 12 articles included in these analyses. The sample sizes in this category ranged widely from 11 to 554 subjects, with most of the studies having relatively small sample sizes (<100). Most of the studies’ subjects were adults and seniors, who were from hospital-based cohorts, followed by population-based databases and general population. Study characteristics, including study design, whether or not applying DNA purification, and whether or not having genotyping and gene expression tests did not vary between small and large sample sized studies. The only tissue used in this group of studies was the whole blood. Most studies (66.7%, 8/12) tested for DNA purification, but not methylation validation. DNA methylation was mostly tested by bisulfite conversion and pyrosequencing. Over half (7/12) of these studies also included genotyping and gene expression in their arrays.

A half of studies (6/12) did not identify significant methylation modifications related to antidepressants use, including the only study on *MAOA*, one of three studies on *SLC6A4*, three of five studies on *BDNF*, and one genome-wide study.^29-34^ One of six studies on *BDNF*, one of three studies on *SLC6A4,* and the only study on IL-6 found that hypermethylations were associated with antidepressant therapy.^22, 35, 36^ Whereas, the only study on *5-HTT* transcriptional control region indicated that its hypomethylation might impair antidepressant response in Caucasian patients with MDD, and one of five studies on *BDNF* promoter region hypomethylations were linked to antidepressant treatment response in remitters compared with non-remitters.^37, 38^ Okada et al.^39^ identified the positive correlation between pre-treatment DNA methylation on *SLC6A4 CpG3* and antidepressants in un-medicated patients. In addition, one study tested the association between methylation modifications and classes of antidepressants, and demonstrated that lithium and valproate tended to decrease, though not significantly, DNA methylation level on *BDNF* promoter, compared with other classes of medications, such as antidepressants and atypical antipsychotics.^29^ Only the small sample-size studies in this group did methylation validation. The only two negative correlations between methylation levels on *BDNF* and *5-HTT* gene and antidepressants were found by studies with relatively small samples.^37, 38^

**References in this appendix**

1. Sabunciyan S, Aryee MJ, Irizarry RA, Rongione M, Webster MJ, Kaufman WE *et al.* Genome-wide DNA methylation scan in major depressive disorder. *PLoS One* 2012; **7**(4)**:** e34451.

2. Byrne EM, Carrillo-Roa T, Henders AK, Bowdler L, McRae AF, Heath AC *et al.* Monozygotic twins affected with major depressive disorder have greater variance in methylation than their unaffected co-twin. *Transl Psychiatry* 2013; **3:** e269.

3. Tseng PT, Lin PY, Lee Y, Hung CF, Lung FW, Chen CS *et al.* Age-associated decrease in global DNA methylation in patients with major depression. *Neuropsychiatr Dis Treat* 2014; **10:** 2105-2114.

4. Uddin M, Koenen KC, Aiello AE, Wildman DE, de los Santos R, Galea S. Epigenetic and inflammatory marker profiles associated with depression in a community-based epidemiologic sample. *Psychol Med* 2011; **41**(5)**:** 997-1007.

5. Choi S, Han KM, Won E, Yoon BJ, Lee MS, Ham BJ. Association of brain-derived neurotrophic factor DNA methylation and reduced white matter integrity in the anterior corona radiata in major depression. *J Affect Disord* 2015; **172:** 74-80.

6. Kim JM, Stewart R, Kang HJ, Kim SW, Shin IS, Kim HR *et al.* A longitudinal study of SLC6A4 DNA promoter methylation and poststroke depression. *J Psychiatr Res* 2013; **47**(9)**:** 1222-1227.

7. Philibert RA, Sandhu H, Hollenbeck N, Gunter T, Adams W, Madan A. The relationship of 5HTT (SLC6A4) methylation and genotype on mRNA expression and liability to major depression and alcohol dependence in subjects from the Iowa Adoption Studies. *Am J Med Genet B Neuropsychiatr Genet* 2008; **147B**(5)**:** 543-549.

8. van der Knaap LJ, van Oort FV, Verhulst FC, Oldehinkel AJ, Riese H. Methylation of NR3C1 and SLC6A4 and internalizing problems. The TRAILS study. *J Affect Disord* 2015; **180:** 97-103.

9. Zhao J, Goldberg J, Bremner JD, Vaccarino V. Association between promoter methylation of serotonin transporter gene and depressive symptoms: a monozygotic twin study. *Psychosom Med* 2013; **75**(6)**:** 523-529.

10. Iga J, Watanabe SY, Numata S, Umehara H, Nishi A, Kinoshita M *et al.* Association study of polymorphism in the serotonin transporter gene promoter, methylation profiles, and expression in patients with major depressive disorder. *Hum Psychopharmacol* 2016; **31**(3)**:** 193-199.

11. Nantharat M, Wanitchanon T, Amesbutr M, Tammachote R, Praphanphoj V. Glucocorticoid receptor gene (NR3C1) promoter is hypermethylated in Thai females with major depressive disorder. *Genet Mol Res* 2015; **14**(4)**:** 19071-19079.

12. Kahl KG, Georgi K, Bleich S, Muschler M, Hillemacher T, Hilfiker-Kleinert D *et al.* Altered DNA methylation of glucose transporter 1 and glucose transporter 4 in patients with major depressive disorder. *J Psychiatr Res* 2016; **76:** 66-73.

13. Zill P, Baghai TC, Schule C, Born C, Frustuck C, Buttner A *et al.* DNA methylation analysis of the angiotensin converting enzyme (ACE) gene in major depression. *PLoS One* 2012; **7**(7)**:** e40479.

14. Haghighi F, Galfalvy H, Chen S, Huang YY, Cooper TB, Burke AK *et al.* DNA methylation perturbations in genes involved in polyunsaturated Fatty Acid biosynthesis associated with depression and suicide risk. *Front Neurol* 2015; **6:** 92.

15. Hohne N, Poidinger M, Merz F, Pfister H, Bruckl T, Zimmermann P *et al.* FKBP5 genotype-dependent DNA methylation and mRNA regulation after psychosocial stress in remitted depression and healthy controls. *Int J Neuropsychopharmacol* 2014; **18**(4).

16. Roy B, Shelton RC, Dwivedi Y. DNA methylation and expression of stress related genes in PBMC of MDD patients with and without serious suicidal ideation. *J Psychiatr Res* 2017; **89:** 115-124.

17. Shi M, Sun H, Xu Y, Wang Z, Cui H, Wang C *et al.* Methylation Status of the Serotonin Transporter Promoter CpG Island Is Associated With Major Depressive Disorder in Chinese Han Population: A Case-Control Study. *J Nerv Ment Dis* 2017; **205**(8)**:** 641-646.

18. Han KM, Won E, Kang J, Choi S, Kim A, Lee MS *et al.* TESC gene-regulating genetic variant (rs7294919) affects hippocampal subfield volumes and parahippocampal cingulum white matter integrity in major depressive disorder. *J Psychiatr Res* 2017; **93:** 20-29.

19. Melas PA, Wei Y, Wong CC, Sjoholm LK, Aberg E, Mill J *et al.* Genetic and epigenetic associations of MAOA and NR3C1 with depression and childhood adversities. *Int J Neuropsychopharmacol* 2013; **16**(7)**:** 1513-1528.

20. Melas PA, Forsell Y. Hypomethylation of MAOA's first exon region in depression: a replication study. *Psychiatry Res* 2015; **226**(1)**:** 389-391.

21. Cordova-Palomera A, Fatjo-Vilas M, Gasto C, Navarro V, Krebs MO, Fananas L. Genome-wide methylation study on depression: differential methylation and variable methylation in monozygotic twins. *Transl Psychiatry* 2015; **5:** e557.

22. Ryan J, Pilkington L, Neuhaus K, Ritchie K, Ancelin ML, Saffery R. Investigating the epigenetic profile of the inflammatory gene IL-6 in late-life depression. *BMC Psychiatry* 2017; **17**(1)**:** 354.

23. Cruceanu C, Kutsarova E, Chen ES, Checknita DR, Nagy C, Lopez JP *et al.* DNA hypomethylation of Synapsin II CpG islands associates with increased gene expression in bipolar disorder and major depression. *BMC Psychiatry* 2016; **16**(1)**:** 286.

24. Chagnon YC, Potvin O, Hudon C, Preville M. DNA methylation and single nucleotide variants in the brain-derived neurotrophic factor (BDNF) and oxytocin receptor (OXTR) genes are associated with anxiety/depression in older women. *Front Genet* 2015; **6:** 230.

25. Meng L, Chen D, Pei F, Hui R, Zheng Y, Chen J. DNA methylation in the norepinephrine transporter gene promoter region is not associated with depression and hypertension. *Clin Exp Hypertens* 2017; **39**(6)**:** 539-545.

26. Kaut O, Sharma A, Schmitt I, Hurlemann R, Wullner U. DNA methylation of DLG4 and GJA-1 of human hippocampus and prefrontal cortex in major depression is unchanged in comparison to healthy individuals. *J Clin Neurosci* 2017; **43:** 261-263.

27. Zhang Y, Chang Z, Chen J, Ling Y, Liu X, Feng Z *et al.* Methylation of the tryptophan hydroxylase2 gene is associated with mRNA expression in patients with major depression with suicide attempts. *Mol Med Rep* 2015; **12**(2)**:** 3184-3190.

28. Kaminsky Z, Payne J. Seeing the future: epigenetic biomarkers of postpartum depression. *Neuropsychopharmacology* 2014; **39**(1)**:** 233-234.

29. Dell'Osso B, D'Addario C, Carlotta Palazzo M, Benatti B, Camuri G, Galimberti D *et al.* Epigenetic modulation of BDNF gene: differences in DNA methylation between unipolar and bipolar patients. *J Affect Disord* 2014; **166:** 330-333.

30. Davies MN, Krause L, Bell JT, Gao F, Ward KJ, Wu H *et al.* Hypermethylation in the ZBTB20 gene is associated with major depressive disorder. *Genome Biol* 2014; **15**(4)**:** R56.

31. Kang HJ, Kim JM, Stewart R, Kim SY, Bae KY, Kim SW *et al.* Association of SLC6A4 methylation with early adversity, characteristics and outcomes in depression. *Prog Neuropsychopharmacol Biol Psychiatry* 2013; **44:** 23-28.

32. Na KS, Won E, Kang J, Chang HS, Yoon HK, Tae WS *et al.* Brain-derived neurotrophic factor promoter methylation and cortical thickness in recurrent major depressive disorder. *Sci Rep* 2016; **6:** 21089.

33. Domschke K, Tidow N, Schwarte K, Ziegler C, Lesch KP, Deckert J *et al.* Pharmacoepigenetics of depression: no major influence of MAO-A DNA methylation on treatment response. *J Neural Transm (Vienna)* 2015; **122**(1)**:** 99-108.

34. Tadic A, Muller-Engling L, Schlicht KF, Kotsiari A, Dreimuller N, Kleimann A *et al.* Methylation of the promoter of brain-derived neurotrophic factor exon IV and antidepressant response in major depression. *Mol Psychiatry* 2014; **19**(3)**:** 281-283.

35. Booij L, Szyf M, Carballedo A, Frey EM, Morris D, Dymov S *et al.* DNA methylation of the serotonin transporter gene in peripheral cells and stress-related changes in hippocampal volume: a study in depressed patients and healthy controls. *PLoS One* 2015; **10**(3)**:** e0119061.

36. Carlberg L, Scheibelreiter J, Hassler MR, Schloegelhofer M, Schmoeger M, Ludwig B *et al.* Brain-derived neurotrophic factor (BDNF)-epigenetic regulation in unipolar and bipolar affective disorder. *J Affect Disord* 2014; **168:** 399-406.

37. Kleimann A, Kotsiari A, Sperling W, Groschl M, Heberlein A, Kahl KG *et al.* BDNF serum levels and promoter methylation of BDNF exon I, IV and VI in depressed patients receiving electroconvulsive therapy. *J Neural Transm (Vienna)* 2015; **122**(6)**:** 925-928.

38. Domschke K, Tidow N, Schwarte K, Deckert J, Lesch KP, Arolt V *et al.* Serotonin transporter gene hypomethylation predicts impaired antidepressant treatment response. *Int J Neuropsychopharmacol* 2014; **17**(8)**:** 1167-1176.

39. Okada S, Morinobu S, Fuchikami M, Segawa M, Yokomaku K, Kataoka T *et al.* The potential of SLC6A4 gene methylation analysis for the diagnosis and treatment of major depression. *J Psychiatr Res* 2014; **53:** 47-53.
